# PARP Inhibition in Prostate Cancer With Homologous Recombination Repair Alterations

**DOI:** 10.1200/PO.21.00152

**Published:** 2021-10-22

**Authors:** Alexander von Werdt, Laura Brandt, Orlando D. Schärer, Mark A. Rubin

**Affiliations:** ^1^ Department for BioMedical Research (DBMR), University of Bern, Bern, Switzerland; ^2^ Institute of Basic Science—Center for Genomic Integrity, Ulsan, South Korea; ^3^ Renaissance School of Medicine at Stony Brook University, Stony Brook, NY; ^4^ Bern Center for Precision Medicine, University of Bern and University Hospital Bern, Bern, Switzerland

## Abstract

**PURPOSE:**

With the broad use of next-generation sequencing assays, it has become clear that mutations in DNA repair genes are more commonly found than previously reported. In advanced prostate cancer patients with *BRCA*1/2 or *ATM* mutations, poly (ADP-ribose) polymerase inhibition (PARPi) causes an increased overall survival advantage compared with patients without these mutations. This review explores the advantages and limitations of PARPi treatment and its use beyond *BRCA*1/2-altered tumors. Furthermore, it discusses the benefits of current biomarkers and what role functional biomarkers and organoids may play in addressing the involvement of homologous recombination repair mutations in tumor development and progression.

**METHODS:**

A systematic review was conducted in MEDLINE, National Library of Medicine, and ClinicalTrials.gov to identify studies published between January 1, 2016, and August 31, 2021. The search strategy incorporated terms for PARPi, BRCA, DNA damage, homologous recombination, organoids, patient-derived organoids, biomarker AND prostate cancer, breast cancer, ovarian cancer.

**RESULTS:**

A total of 261 records remained after duplicate removal, 69 of which were included in the qualitative synthesis.

**CONCLUSION:**

To improve the outcome of targeted therapy and increase sensitivity of tumor detection, patients should be repeatedly screened for DNA repair gene alterations and biomarkers. Future clinical studies should explore the use of PARPi beyond *BRCA*1/2 mutations and focus on finding new synthetically lethal interactions.

## INTRODUCTION

The promise of precision oncology is to identify the right drug, for the right patient, at the right time. Targeted cancer therapy has demonstrated significant success in this regard. The paradigm of treating the so-called driver mutation is successful but with important caveats. First, not all cancers have well-defined and clear driver mutations. Second, even when the driver mutation is known, it may not be targetable. Third, resistance to therapy is more the rule than the exception. Finally, the response to single-agent targeted therapy also depends on the context and type of cancer.

**CONTEXT**

**Key Objective**
With the broad use of next-generation sequencing assays, it has become clear that mutations in DNA repair genes are more commonly found than previously reported. In advanced prostate cancer patients with *BRCA*1/2 or *ATM* mutations, poly (ADP-ribose) polymerase inhibition (PARPi) causes an increased overall survival advantage compared with patients without these mutations.
**Knowledge Generated**
This review explores the advantages and limitations of PARPi treatment and its use beyond *BRCA*1/2-altered tumors. Furthermore, it discusses the benefits of current biomarkers and what role functional biomarkers and organoids may play in addressing the involvement of homologous recombination repair mutations in tumor development and progression.
**Relevance**
To improve the outcome of targeted therapy and increase sensitivity of tumor detection, patients should be repeatedly screened for DNA repair gene alterations and biomarkers. Future clinical studies should explore the use of PARPi beyond *BRCA*1/2 mutations and focus on finding new synthetically lethal interactions.

DNA damage response is vital to a cell's survival. Insufficient response to DNA damage has been shown to contribute to the development of most, if not all, cancers in humans.^1^ Beyond the well-known canonical recurrent driver mutations, targetable genes also have noncanonical mutations, which may have clinical significance. Therefore, an improved understanding as to how to predict which patients will respond to therapy across a wide variety of cancer types and molecular alterations is urgently needed. In this review, we will focus on mutations in homologous recombination repair (HRR) genes and the use of poly (ADP-ribose) polymerase inhibitors (PARPi).

**FIG 1.**
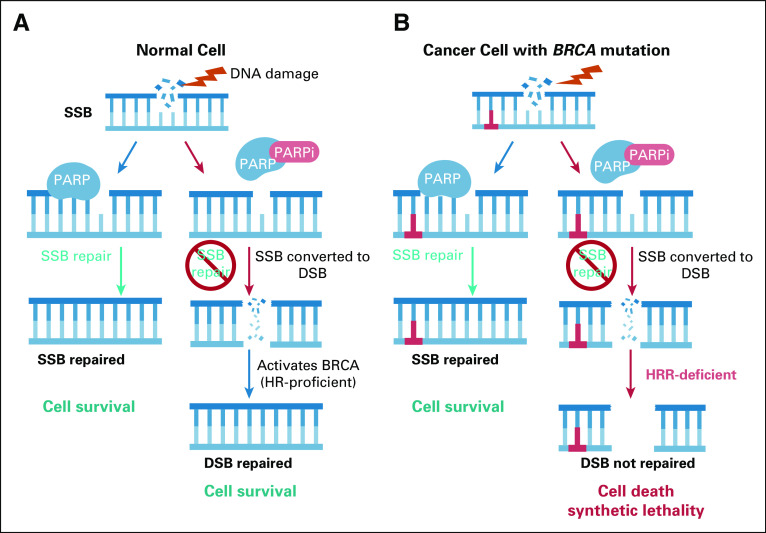
Cell response to DNA damage with and without *BRCA* mutation and when treated with PARPi. (A) Normal cell: (left) If a normal cell incurs DNA damage, PARP binds to the damaged site and recruits DNA repair proteins and repairs the broken DNA strand; (right) if PARP is inhibited, *BRCA* will be activated and the cell survives. Both these mechanisms lead to cell survival. (B) A cancer cell harboring a *BRCA* mutation does not activate downstream effectors. The PARP enzyme is still functional and can assist in the repair process. (left) The cancer cell's replication and survival is then ensured. (B) When PARP is inhibited in a cancer cell harboring *BRCA* mutations, synthetic lethality occurs (right). The cell cannot survive and dies. DSB, double-strand breaks; HRR, homologous recombination; PARP, poly (ADP-ribose) polymerase; PARPi, poly (ADP-ribose) polymerase inhibition; SSB, single-strand breaks.

The two most common types of DNA damage are double-strand breaks (DSBs) and single-strand breaks (SSBs). Each have individual repair pathways. In the example of SSB, poly (ADP-ribose) polymerase (PARP) binds to the SSB and activates its catalytic activity, with the parylation of itself and surrounding proteins initiating break repair^2^ (Fig 1A). DSBs are either repaired through HRR or nonhomologous end joining (NHEJ).^3^ NHEJ is used mainly in the G1 phase and reconnects the broken ends but may induce deletions and insertions and therefore is more error-prone.^4^ HRR uses the homologous chromosome in S/G2 as a template to recreate an exact replica.^4^ BRCA is a tumor suppressor and crucial for HRR. If there is a DNA DSB, BRCA will recruit proteins like RAD51 to repair the breakage^5^ (for a detailed review of HRR, see the study by Li and Heyer). Despite mutations in genes, such as *BRCA* and *ATM*, cancer cells are able to avoid cell death by switching to alternative pathways.^6,7^

Mutations in *BRCA1* and *BRCA2* (*BReast CAncer genes 1* and *2*) and the association with breast cancer were first discovered by teams of researchers led by Mary-Claire King (*BRCA1*) in 1990^8^ and Alan Ashworth and Sir Mike Stratton (*BRCA2*) in 1995.^9^ When *BRCA* is mutated, the HRR repair cascade is interrupted, leading to an HRR defect that results in a failure to correctly repair DNA DSBs^10^ (Fig 1B). Screenings for *BRCA1/2* mutations were the first genetic tests to assess a cancer risk.^11^ In 2003, King et al^12^ discovered that women who had a germline *BRCA* mutation had a 50%-80% increased risk of developing breast cancer and, depending on whether they had a *BRCA1* or *BRCA2* mutation, a 10%-40% increased risk of ovarian cancer development. By analyzing 22 studies with more than 8,000 patients with either breast or ovarian cancer, Anoniou et al confirmed King's findings in 2003.^13^

What occurs when *BRCA* is mutated and no longer able to express and repair DSBs correctly? Toxic lesions arise at much lower frequency in the presence of PARP, so there is less of a need for BRCA*1/2* activity. This creates a dependency on compensatory repair pathways, making PARP essential for *BRCA1/2*-mutant tumor and an opportunity for therapeutic inhibition.

## METHODS

The following databases have been used for a systematic search: National Library of Medicine, ClinicalTrials.gov, MEDLINE (Ovid) database, and PubMed. The search strategy incorporated terms for PARPi, BRCA, DNA damage, homologous recombination, organoids, patient-derived organoids (PDOs), biomarker AND prostate cancer, breast cancer, ovarian cancer. Search for new data was limited to studies published between 2016 and 2021. A total of 262 records were identified, as well as 16 additional records through other sources (experts and opinion leaders in the field). After duplicate removal, 261 records remained, out of which 69 were included in the qualitative synthesis. This study followed the Preferred Reporting Items for Systematic Reviews and Meta-Analyses guideline.

## RESULTS

### Discovery and Targeting of PARP: Exploiting Synthetic Lethality

PARP is an enzyme that functions as a molecular sensor, which recognizes and binds to DNA SSB and is involved in several cellular processes, including the DNA damage response pathway.^14^ An SSB provides a specific docking site for the zinc finger domain of PARP. This initial step allows PARP to activate its catalytic ADP-ribosyltransferase activity.^14^ ADP-ribosyltransferase recruits substrate proteins and DNA repair effectors.^15^ The conceptual framework of how PARPi interacts with *BRCA* mutations is called synthetic lethality (SL) (for a detailed review on SL and PARPi, see the study by Ashworth et al and Lord et al). SL is the combined loss of both components that result in cell death because of the interdependent and/or compensatory nature of the pathways. SL is built around the idea that mutations, in this example, genes involved with HRR, such as *BRCA* and *ATM*, are advantageous to the tumor cell. If a second component like PARP is inhibited, the mutated HRR gene and the second component generate a tumor-specific vulnerability (Fig 1B). PARPi traps PARP on DNA, resulting in the stalling of the replication fork.^15^ Generally, the stalled replication fork activates HRR, which involves *BRCA1/2*, to repair and reactivate the replication fork.^15^ A defect in HRR leads to the recruitment of other less effective repair proteins.^15^ Although normal cells with functional BRCA are able to repair the DSBs accurately, cancer cells deficient for BRCA use microhomology-mediated end joining, a more error-prone repair, which can lead to high levels of genomic instability and cancer cell death.^14^ Therefore, PARPi specifically targets cancer cells. When both *BRCA1* and *BRCA2* alleles are present, they show a selective toxicity to PARPi compared with when one wild-type allele is present.^14,16^ The discovery of this vulnerability led to significant efforts to develop PARPi to treat cancer patients with mutations in genes involved in the DSB repair pathway.

Another example for SL in the context of deficient HRR is the DNA repair enzyme polymerase Q (POLQ).^17^ POLQ has been shown to be overexpressed and upregulated in numerous cancer types including breast cancer and PCa.^17^ A synthetic lethal interaction has been observed between POLQ inhibition and *BRCA1/2* mutations.^17^ The development of POLQ inhibitors for the treatment of patients with *BRCA* mutations is currently ongoing and will hopefully reach clinical trials soon.

**FIG 2.**
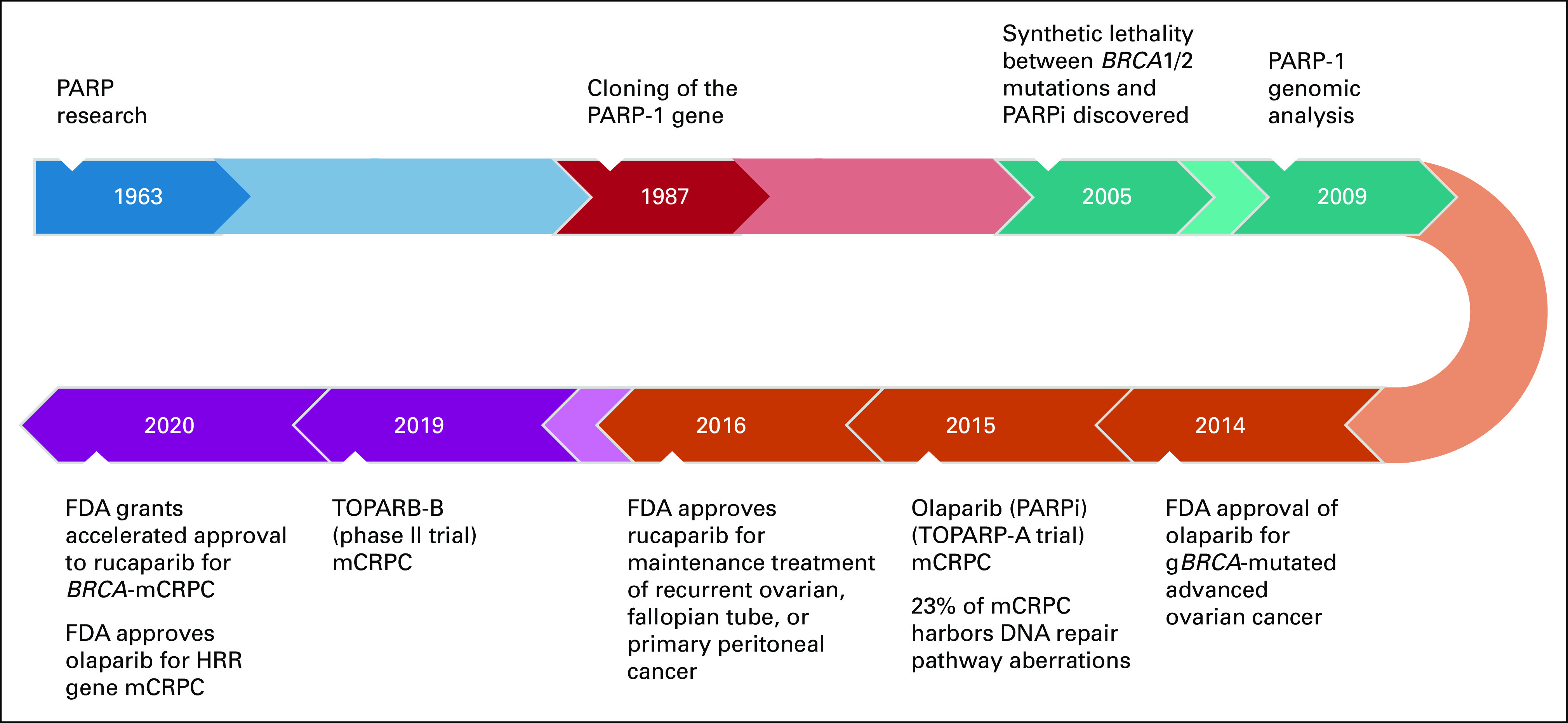
Timeline of PARPi approval for mCRPC illustrating PARP milestones and prostate cancer milestones. FDA, US Food and Drug Administration; HRR, homologous recombination repair; mCRPC, metastatic castration-resistant prostate cancer; PARP, poly (ADP-ribose) polymerase; PARPi, poly (ADP-ribose) polymerase inhibition.

Initially, PARPi was targeted for hereditary breast and ovarian cancer and individuals with germline *BRCA* mutations. On the basis of clinical experience with hereditary cancers, the major susceptibilities were considered breast cancer, ovarian cancer, and endometrial cancer and that those patients would most benefit from a PARPi treatment. On the grounds that PARPi specifically targets tumor cells, the US Food and Drug Administration (FDA) has approved several PARPis for the treatment of several types of cancers. Ovarian cancer was the first in 2014, followed by breast cancer in 2018. Most recently, in May 2020, the FDA and in November 2020, the European Medicines Agency approved PARPi for the treatment of PCa. From the time of PARP discovery, in the late 1960s, to the first FDA approval for use in 2014, it took roughly 45 years (Fig 2). Since targeting the HRR pathway is a relatively novel approach, long-term effects have not been identified and need further exploration.^18^

In 1997, genetic analysis of patients with PCa from Iceland with positive *BRCA2* family background showed that approximately 66% of the cases had *BRCA2* mutations.^19^ This was the first time that *BRCA2* was associated with PCa, thereby providing an important link between breast or ovarian cancer and PCa. In 2009, Fong et al^20^ published their results of the first PARPi trial that was enriched for patients with cancer who had *BRCA1 and BRCA2* mutations. Tumor types included ovarian, breast, melanoma, sarcoma, and prostate. Patients who had a *BRCA* mutation showed a higher antitumor activity than patients without *BRCA* mutations.^20^

Next-generation sequencing studies successfully identified frequent mutations in multiple cancer types and genes involved in HRR that go beyond *BRCA1/2*. In 2013, a retrospective next-generation sequencing analysis, of metastatic castration-resistant prostate cancer (mCRPC) samples, discovered an alteration frequency in *ATM* of 8%.^21^ In 2015, Robinson et al^22^ reported HRR defects in 20% of patients with advanced PCa. A follow-up study by Pritchard et al^23^ confirmed a 20% frequency of HRR defects, either somatic or germline, in several cohorts consisting of more than 700 men without known hereditary cancer syndromes. The implication from this study was that patients with advanced PCa harbor frequent HRR defects, making them potential candidates for PARPi regardless of family history.

In an early PARPi trial in 2013, only 3 of 25 patients with PCa responded to the therapy.^24^ There was no patient selection on the basis of genetic alterations as to who would most likely respond. Several factors account for expanded interest in investigating HRR alterations in PCa: the high frequency of HRR mutations in multiple cancers including PCa^21^ combined with the knowledge that there is a significantly higher risk of developing PCa with *BRCA2* mutation (8.6×) and the fact that *BRCA2* mutation is more commonly found in patients with PCa.^25^ PARPi has shown clinical benefits in PCa, breast cancer,^26^ and ovarian cancer,^27^ and more clinical trials are currently ongoing (Tables 1 and 2).

**TABLE 1.**
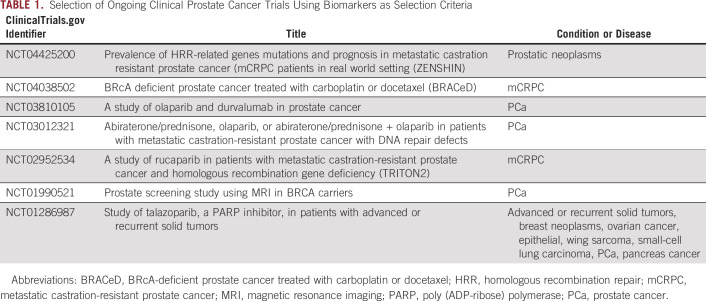
Selection of Ongoing Clinical Prostate Cancer Trials Using Biomarkers as Selection Criteria

**TABLE 2.**
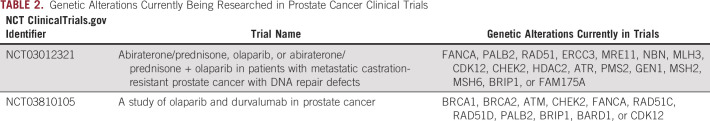
Genetic Alterations Currently Being Researched in Prostate Cancer Clinical Trials

Once the significance of HRR mutations was discovered, HRR mutation patient–enriched trials were able to research targeted treatment not only in PCa but also in a variety of different cancer types.

Although current PARPi trials are primarily focusing on *BRCA1* and *BRCA2* mutations, cancers that do not have *BRCA* mutations may still be vulnerable to PARPi therapy, as other alterations such as *ATR*, *PALB2*, and the members of the *FANC* gene family^15^ can confer a *BRCA*-like status.^15^

### PARPi Resistance

**FIG 3.**
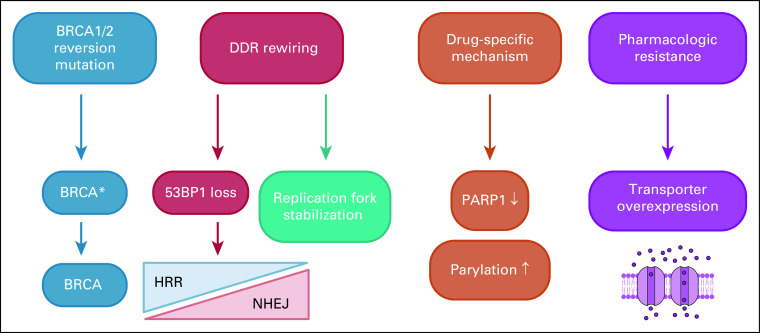
PARPi resistance mechanisms. Multiple mechanisms of resistance against PARPi have been identified. (1) Restoration of HRR by BRCA reversion mutations which re-establish the ORF of BRCA is the most commonly observed mechanism of PARPi resistance. BRCA (*BReast CAncer gene*) reversion mutations restore the open reading frame of *BRCA1/2* and can be identified in circulating cfDNA.^30^ (2) Rewiring of the DNA damage response network via 53BP1 loss or replication fork stabilization can cause reactivation of the HRR pathway. It has been shown that loss of 53BP1-RIF1-REV7-Shieldin signaling, which is involved in NHEJ, in *BRCA1*-deficient cells reactivates resection, rescues HRR activity, and thereby causes PARPi resistance. Mutations in TMEJ-specific signals are often present in *BRCA*-mutated, PARPi-resistant cells.^17^ *53BP1* loss and a depletion of the TMEJ gene *POLQ* display synthetic lethality with each other and have shown to enhance sensitivity of HRR-deficient cells to PARPi.^29^ (3) Additionally, drug-specific mechanisms such as downregulation of PARP1 expression and increased parylation as well as (4) overexpression of the drug transporter and thereby insufficient accumulation of the PARPi can restore BRCA signaling. DDR, DNA damage response; HRR, homologous recombination repair; NHEJ, nonhomologous end joining; PARPi, poly (ADP-ribose) polymerase inhibition; TMEJ, theta-mediated end joining.

Despite the initial efficacy of PARPi for the treatment of *BRCA*-mutated tumors, therapy resistance is commonly observed.^28^ The restoration of HRR is the most commonly observed mechanism of PARPi resistance. Restoration happens mostly through *BRCA1/2* repair or by changes in the DNA damage response pathway, for example, the loss of 53BP1.^29^ In 2008, the impact of reversion mutations in HRR genes, specifically *BRCA1/2* on the restoration of HRR signaling (Fig 3) and increased resistance to PARPi treatment, has been discovered.^30-32^ *BRCA* reversion mutations (most commonly < 100 bp deletions) restore the open reading frame of *BRCA1/2* and can be identified in circulating cell-free DNA.^30^ Cell-free DNA can be isolated from the blood and used to monitor *BRCA* reversion mutations. This represents a minimally invasive assay that can be used to detect reversion mutations, identify PARPi effectiveness throughout treatment, and help guide physicians to find the next adequate treatment.^30^ Additionally, many reversion mutations are predicted to create tumor neoantigens, which could potentially be helpful in targeting resistance.^31^ In addition to *BRCA1/2*, secondary mutations in other HRR genes, such as *RAD51C* and *RAD51D*, are shown to be leading factors in restoring a functioning HRR pathway.^33^ Sequencing of HRR pathway genes in 12 biopsies from the *ARIEL 2 phase II* study, which aimed to identify patients with ovarian cancer who were likely to respond to PARPi treatment, identified primary and secondary mutations in *RAD51C* and *RAD51D*. Although primary mutations in these two genes showed SL sensitivity to PARPi, secondary mutations correlated with acquired PARPi resistance.^34^

Besides the restoration of HRR pathway members, rewiring of the DNA damage response network can also cause reactivation of the HRR pathway (Fig 3). It has been shown that loss of 53BP1-RIF1-REV7-Shieldin signaling, which is involved in NHEJ, in *BRCA1*-deficient cells reactivates resection, rescues HRR activity, and thereby causes PARPi resistance. Mutations in theta-mediated end joining–specific signals are often present in *BRCA*-mutated, PARPi-resistant cells.^17^ 53BP1 loss and a depletion of the theta-mediated end joining gene *POLQ* display SL with each other and have shown to enhance sensitivity of HRR-deficient cells to PARPi.^29^

Unconnected to their involvement in HRR, *BRCA1/2* support genomic stability by maintaining replication fork integrity (Fig 3) under replicative stress.^35^ Studies by Ray Chaudhuri et al^35^ showed that PARPi resistance was directly connected to replication fork stabilization and prevention of fork collapse.

PARPi resistance can also arise because of drug target–related mechanisms (Fig 3) such as alterations in PARP1 expression or parylation.^29^ PARP1 is a key mediator in the effectiveness of PARPi therapy. The reduction or downregulation of PARP1 combined with the restoration of BRCA activity can lead to PARPi resistance.^29^ *In vitro* experiments showed that increased parylation seemed to restore PARP function by inactivating a component of the PARP mechanism, PARG in the presence of PARPi.^29^

Finally, pharmacologic resistance (Fig 3) to PARPi can be acquired via overexpression of the ATP-binding cassette drug transporter and results in insufficient accumulation of the cancer drug.^29^

Although the mechanistic insights into the molecular vulnerabilities that lead to PARPi resistance are often still unclear, these findings are important to guide the development of alternative therapies and to overcome PARPi resistance.

### HRR Biomarkers in Advanced PCa

To improve treatment outcome, it is important to find suitable biomarkers to determine the effectiveness of HRR-targeted treatments. A subset of patients with mCRPC has shown a strong response to PARPi as first noted by Mateo et al^36^ who described that 30% of the PARPi-treated mCRPC cases responded to the therapy.

In a review of clinical PCa trials (Table 3), which included a screening for prespecified DNA mutations, usually involving HRR biomarkers, it was observed that 88% of the samples tested came back with a reported biomarker status, of which 17% showed mutations in the selected genes. The data demonstrates that 10.5% of patients enrolled were found to have genetic alterations (ranged from 1% to 29.5%). These numbers show that only about 10% of patients originally enrolled could be eligible for a specific biomarker study. This means that the majority of patients tested were not eligible for the clinical trial. This lack of specificity is a significant problem with biomarker trials. Despite all these limitations, a successful PCa biomarker study was published in 2020. The recently published PROfound study, the first major biomarker study in PCa, revealed significant findings in patients with *BRCA1*, *BRCA2*, and *ATM* mutations. Of 4,425 patients enrolled in the study, 778 (17.6%) showed alterations in at least one the of 15 predefined HRR genes.^37^

**TABLE 3.**
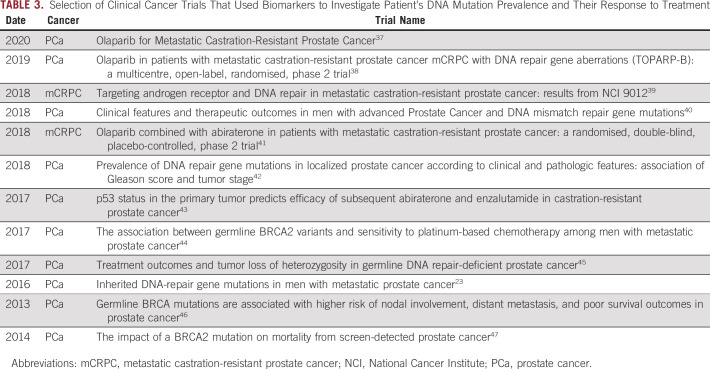
Selection of Clinical Cancer Trials That Used Biomarkers to Investigate Patient's DNA Mutation Prevalence and Their Response to Treatment

The PROfound study showed that there was an increase in progression-free survival and overall survival (OS) among cohort A (which included PCa patients with at least one of the following mutations: *BRCA1*, *BRCA2*, or *ATM* mutations). Cohort B was made up of patients with at least one of the 12 prespecified gene mutations, such as *FANCL* and *PALB2*. Different levels of antitumor activity were observed depending on HRR alterations. Patients with *BRCA1*, *BRCA2*, and *ATM* alterations presented the greater advantage in therapeutic benefit by responding better to therapy and having a better OS.^48^ Patients with long-tail HRR alterations, for example, *FANCL* or *RAD51C*, did not show significant clinical benefit.^48^ By analyzing the impact of previous therapies that patients had undergone, Hussain et al^48^ found that patients who harbored *ATM* alterations and were previously treated with taxanes showed an increased benefit in OS. Moreover, it has been reported that patients with *BRCA2*-mutated PCa show a superior response to PARPi than *BRCA1*-mutated patients.^49,50^ Markowski and Antonarakis^50^ collected data from five *PARPi* studies, which show the differential response in *BRCA1*- compared with *BRCA2*-mutated patients. Pan-cancer analysis of *BRCA1* and *BRCA2* alterations has revealed that genome-wide loss of heterozygosity (gLOH) is a possible marker for PARPi sensitivity.^51^ An increase in gLOH was seen across different cancer types, including PCa, that had biallelic *BRCA1/2* alterations,^51^ and gLOH presents a possible biomarker to evaluate PAPRi sensitivity.

A recent study used CRISPR-Cas9 screens to identify PARPi sensitivity marker and found that alterations in the genes encoding the RNase H2 enzyme complex (*RNASEH2A*, *RNASEH2B*, and *RNASEH2C*) cause PARPi sensitivity of cells via impaired ribonucleotide excision repair.^52^

Molecular signatures, for example, homologous recombination deficiency (HRD) scores, can help to guide treatment decisions. Lotan et al^53^ assessed HRD scores in patients with primary PCa and found that germline *BRCA2*–mutated patients had the highest HRD score, followed by *ATM* and *CHEK2* alterations. Although clinical studies have shown a better PARPi response in *BRCA2*-mutated PCa compared with *ATM* and *CHEK2* alterations,^37^ Lotan et al^53^ showed the same correlation with higher HRD scores in the respective HRR gene mutations. A positive correlation has been found between a higher HRD score and a better clinical outcome in patients with PCa receiving PARPi treatment.^53^ These findings support further exploration in functional HRR assay research and testing for clinical purposes.

A possible exclusion criterion for using PARPi, *PPP2R2A* alterations (this enzyme is associated with cell growth and is considered long tail),^54^ was also discovered. The finding of the link between *PPP2R2A* mutation status and PARPi opens up the field for further investigations of possible exclusion criteria for PARP therapy. Also, retrospective studies have presented evidence for *CDK12* alterations as another non–PARPi-sensitizing marker.^55,56^ A multicenter study did not find any meaningful change of clinical outcome in a PCa cohort that was given PARPi with *CDK12* alterations.^55^ Patients with *CDK12*-mutated PCa instead show potential sensitivity to PD-1 inhibitors.^55^

Interestingly, studies using PCa cell lines have shown that ATM loss does not increase sensitivity to PARPi but rather to ataxia telangiectasia and RAD3-related (ATR) inhibition^57^ and the first clinical trial of the ATR inhibitor BAY 1895344 confirmed antitumor activity in ATM-deficient cancer patients.^58^ In addition, ATR inhibitors showed antitumor activity in cancers with *BRCA1* mutations that were resistant to PARPi.^58^ Therefore, it is important to carefully analyze the mutation status of the individual HRR genes to make a personalized treatment decision.

To increase sensitivity of tumor detection and to improve the outcome of targeted therapy strategies, patients should be screened for several biomarkers. To investigate the genetic risk of cancer, there is a gene panel called B.O.P. (*Breast*, *Ovarian*, and *Prostate*), which consists of targeting 87 genes. These 87 genes have been suggested, predicted, or clinically proven to be associated with breast, ovarian, and/or PCa risk.^59^ By acknowledging that not all HRR-mutated tumors can be categorized and viewed the same,^18^ preclinical trials should create viable models by functionally analyzing potential biomarkers. Furthermore, the successful development of targeted therapy is dependent on establishing reliable assays of response and resistance.^18^

### Combination Therapy

Understanding and overcoming acquired resistance to PARPi is essential for continuing research and optimization of PARPi therapy. Combination strategies, exploring the combination of PARPi with non–DNA-damaging agents^60^ or directly targeting PARPi-resistant mechanisms,^60^ are currently being explored in clinical trials. Preclinical studies have explored SL interactions with other DNA repair genes such as *ATR*, *PALB2*, and members of the *FANC* gene family,^15^ which can confer a *BRCA*-like status.^15^ Preclinical trials have shown positive outcomes and sensitivity when combining PARPi and ATR inhibitors in human ATR–deficient lung, prostate, and pancreatic cancer cells.^61^ Therefore, future trial designs should explore new combinations of therapies for long-tail alterations to detect new SLs.

Furthermore, the interaction of hormone receptor signaling and PARPi is being explored.^62^ There are currently four phase III clinical trials exploring the combination of PARPi and AR pathway targeting (TALAPRO2 [NCT03395197], CASPAR [NCT04455750], PROpel [NCT03732820] and MAGNITUDE [NCT03748641]).^63^

## DISCUSSION

Given the limitations of biomarker testing for targeted therapy discussed above, the use of preclinical models for screening anticancer drugs and evaluating drug responses could help to improve patient response and to guide clinical decisions. One option are patient-derived organoids (PDOs).

Organoids are commonly derived through stem cells, self-organizing, three-dimensional structures, which mimic the in vivo tissue ex vivo^64^ (for a detailed review on human organoids, see the study by Kim et al). PDOs are cells that are extracted from the patient and grown ex vivo.^65^ As PDOs reflect the architecture, geno- and phenotype of a patient's tumor, they are a better model than cell lines to aid the development of new treatments and may be used for predictive tests for how the patient may respond to different drugs. There are, however, some limitations to the widespread use of PDOs, such as the absence of the microenvironment, loss of the heterogeneity from the original tumor, and prolonged organoid culture that can lead to genomic drifts, which then prevents them from being an exact replica of the patient's tumor. Pauli et al^66^ also discovered that the amount of fresh tissue needed to extract enough viable tumor cells is a technical limitation, which so far has prevented the successful use of organoids for functional testing. Finally, depending on the cancer type, only a small fraction of patient-derived organoids can be established.^66^ Despite the limitations described above, functional organoid lines have been established for a few cancers, including PCa^67^ and ovarian cancer.^67^

One example where murine prostate organoids have been used was a study by Boysen et al in 2015. They showed that SPOP, the most commonly mutated gene in both clinically localized and metastatic PCa, modulates DNA DSB repair and that organoids with *SPOP* mutation show increased levels of genomic instability.^68^ Alterations in *SPOP* cause impaired HRR similar to tumors with *BRCA1* mutations.^68^ Further experiments presented evidence that *SPOP* mutations sensitize to PARPi,^68^ but no clinical trials have been conducted to explore the relevance of these preclinical findings.

Hill et al used organoids derived from high-grade serous ovarian cancers to develop a functional test. They showed that *BRCA1/2* or *FANCA* mutations alone are not enough to secure sensitivity to PARPi.^67^ Only 6% of the organoids tested had functional HRR defects. This suggests that regardless of the amount of detected mutation frequency, there are a significantly larger number of cases or organoids (in this case) that potentially could be treated with the proper inhibitor and that PARPi is not the only drug type that should be considered.

Moving forward, PDOs can be included to guide patient treatment and drug development and be used for rapid drug screening. Importantly, PDOs could also be used to study biomarkers for preventive studies.

In conclusion, BRCAness concerns a larger group than just the initial concept. Most people originally thought of this as breast and ovarian cancer syndrome, but in fact, it expands to other cancers, including PCa.

Mutations in the DNA repair pathway are more frequent than anticipated, not always family-related as expected, and demonstrate a higher therapeutic benefit in a wider scope of cancers. Even if the focus rested on *BRCA* and *ATM*, other HRR mutations could still be relevant.

Contemplating the sensitivity and specificity of a biomarker, one must also consider the scope. By only selecting cases with *BRCA2* alterations, one might have a lot of patients who respond but might also miss a lot of patients who would have responded had there been a wider selection of alterations.

One important aspect to remember about PARPi is that the alignment of a biomarker to a response is not a perfect correlation.

There are some established biomarkers that can be used to identify patients with a higher cancer risk and that are suitable for PARPi, like BRCA*1/2*. But what about patients with long-tail mutations or functionally disruptive HRR? How will those be measured? The future of treatment choice on the basis of biomarkers is a combination of genomic analysis and functional testing to identify targetable DNA defects. Organoids could be very useful in this quest to find new SLs. Response to therapy can be studied in functional organoids, mimicking a disease with specific mutations, without placing the patient at risk. The long-term benefits of increasing genetic testing need to be investigated on a broad scale. Analysis of early one-time screening compared with long-term treatment costs and consideration of the benefits to family members of learning if they have a genetic predisposition cannot be ignored. This presents a substantial justification to reconsider and change current genetic testing guidelines.^69^

Precision medicine is exactly what the term suggests, medicine tailored for the genetic makeup of an individual. Patient treatment is optimized by addressing problems like resistance and lack of response to conventional and targeted therapy. Clinical trials with PARPi and corresponding biomarkers are currently ongoing. HRR alterations have been shown to be useful biomarkers. Precision medicine in the future will depend on biomarkers that can precisely predict therapeutic outcome. Furthermore, the use of combination therapy through research and application aids the detection of new SL combinations and the fight against PARPi resistance seen in some patients.
